# Exploring Immersive Virtual Reality as an Approach to Improve School Participation–Related Constructs in Children With ADHD

**DOI:** 10.1111/cch.70328

**Published:** 2026-08-11

**Authors:** Barkın Köse, Emine Cansu Güler, Güleser Güney Yılmaz, Müberra Tanrıverdi, Dana Anaby

**Affiliations:** ^1^ Department of Occupational Therapy Faculty of Gülhane Health Sciences, University of Health Sciences Turkey Ankara Turkey; ^2^ School of Physical and Occupational Therapy Faculty of Medicine and Health Sciences, McGill University Montreal Canada; ^3^ Department of Occupational Therapy Faculty of Health Sciences, Kütahya University of Health Sciences Kütahya Turkey; ^4^ Department of Occupational Therapy Faculty of Health Sciences, Hacettepe University Ankara Turkey; ^5^ Department of Physiotherapy and Rehabilitation Faculty of Health Sciences, Bezmialem Vakıf University İstanbul Turkey

**Keywords:** ADHD, immersive virtual reality, school participation

## Abstract

**Background:**

School participation is frequency and involvement from person–environment transactions, not diagnosis, per the International Classification of Functioning, Disability and Health (ICF) and the family of participation‐related constructs (fPRC). In this framework, the environmental and child determinants of school participation (e.g., school routines and peer/teacher context; self‐regulation, activity competence and preferences) interact bidirectionally to shape everyday participation. However, many interventions still target isolated impairments, overlooking coordinated changes in capacities and context. Grounded in this contemporary view, this study aimed to investigate the impact of an immersive virtual reality (IVR) intervention on school participation–related constructs in children with ADHD.

**Methods:**

The study included 92 children aged between 7 and 12 years diagnosed with ADHD. Participants were randomly assigned into intervention (*n* = 46) and control (*n* = 46) groups. Both groups completed the School Participation Questionnaire (SPQ) and Bruininks–Oseretsky Test of Motor Proficiency Test 2 Brief Form (BOT2‐BF) assessment prior to the intervention. The intervention group received an IVR intervention program twice a week for 8 weeks. During this period, the control group did not receive additional therapy. At the end of the 8 weeks, the SPQ was readministered to both groups.

**Results:**

Baseline characteristics showed no significant differences in SPQ and BOT2‐BF results between groups, confirming homogeneity prior to intervention. Following the intervention, the study group demonstrated significant improvements across all domains of the SPQ (doing, being, symptoms and environment), with large effect sizes for SPQ total score (*d* = 0.978) and subdomains (*d* = 0.452–0.910). In contrast, the control group showed no improvements and even declines in subdomains. Post‐intervention, between‐group comparisons revealed significant differences favouring the study group across all domains (*p* < 0.001), with large effect sizes (*d* = 0.878–1.165).

**Conclusions:**

Findings suggest that the IVR program was associated with improvements in teacher‐rated environmental and child determinants of school participation (SPQ domains) in children with ADHD.

## Summary

- Immersive virtual reality (IVR) offers a promising alternative for supporting children with ADHD in managing school‐related participation challenges.
- Incorporating supportive technologies like IVR into school settings could be considered as part of a broader strategy to assist neurodiverse students.
- The findings align with the fPRC and ICF frameworks, illustrating the importance of viewing school participation as a result of person‐environment interactions.
- These findings encourage further investigation into how immersive tools can be integrated into standard occupational therapy protocols for school‐aged children.

## 1 Introduction

Attention‐deficit/hyperactivity disorder (ADHD) is a neurodevelopmental condition characterized by developmentally inappropriate levels of inattention, impulsivity and hyperactivity (Edition F 1980). These core symptoms, including difficulties with attention, concentration and executive functioning, can negatively impact children's participation, especially when supports within the environment are limited (Miller et al. 2012). Existing research indicates that, compared to their typically developing peers, children with ADHD participate less frequently and demonstrate lower levels of involvement across home, school and community contexts (Bedell et al. 2013; Coster et al. 2013; Law et al. 2013). Specifically, these children face distinct challenges in classroom activities, peer interactions outside of class, tasks with high cognitive demands, sensory experiences and the maintenance of safety (Kara et al. 2025).

School participation can be understood through two parts: frequency (being there; how often) and involvement (the quality of the experience while attending) (Imms et al. 2016; Imms et al. 2017). These dimensions are shaped by environmental and child determinants of school participation. As proposed by Maciver et al., while child determinants include identity/engagement, doing/competence and day‐to‐day symptoms, environmental determinants also encompass school structures/routines, adult practices, peer supports, spatial access/sensory fit and the availability of classroom objects and tools (Imms et al. 2017; Maciver et al. 2022). Additionally, motivation, interests, functioning, perceived enjoyment and competence, self‐efficacy, emotional stability and fewer behavioural problems are among the child‐related determinants influencing both children's engagement in school activities and the sustainability of their participation (Lam et al. 2016; Maciver et al. 2019). Environmental determinants such as school routines and rules, sensory load (e.g., noise and lighting), overall accessibility, teacher support, positive teacher–student relationships, peer acceptance and friendship support are among the factors influencing school participation (Kara et al. 2025; Saracostti et al. 2024; Saracostti et al. 2025; Martins et al. 2022). School‐focused measures aligned with this framework, such as the School Participation Questionnaire (SPQ), along with participation indices that distinguish between frequency and involvement, such as the Participation and Environment Measure for Children and Youth (PEM‐CY), offer robust tools for evaluating intervention outcomes (Maciver et al. 2022; Coster et al. 2012).

Schools are essential contexts for the development of academic, social and emotional skills; consequently, a child's level of engagement serves as a significant determinant of both personal outcomes—such as peer relationships and emotional health—and academic achievement (Vaz et al. 2015; Simon‐Dack et al. 2016). It is crucial to assess the factors influencing participation within natural environments like the school setting to develop comprehensive programs that address participation challenges (Anaby et al. 2019; Özbay and Kayhan 2024). Various approaches, including pharmacological, nonpharmacological and combined treatments, have demonstrated beneficial effects on ADHD symptoms and academic functioning. Furthermore, school‐based strategies—ranging from organizational skills training and behavioural classroom interventions to psychosocial supports and technology‐based interventions like virtual reality (VR)—have proven effective in enhancing overall participation (Richardson et al. 2015; Dobrean et al. 2018; Mattingly and Young 2021; Wong et al. 2023).

VR is a technology that simulates real or imagined settings—such as a classroom—enabling users to interact within three‐dimensional environments and respond as they would in real‐life situations (Schwebel et al. 2008; Reid 2002). Immersive VR (IVR), which provides a fully engaging, realistic and interactive experience, is among the most widely utilized forms of VR due to its ability to enhance user experience, increase enjoyment and motivation and provide an opportunity for active participation (Nolin et al. 2016; Parsons et al. 2007). Lobidi et al. (2024) examined the impact of a VR intervention on learning outcomes in children with ADHD, suggesting that such interventions may improve academic performance by enhancing attention and role engagement (Alobidi et al. 2024). Similarly, technology‐based interventions have been shown to potentially strengthen attention, working memory and self‐regulation skills, while supporting academic competencies in areas such as reading and mathematics (Abidali et al. 2025; Liu et al. 2020; Jensen and Konradsen 2018). IVR, with its ability to provide a realistic environment, has the potential to enhance factors influencing children's school performance (Liu et al. 2020). In this context, IVR can target child‐related determinants of school participation, such as executive functions, self‐regulation, emotional regulation, motivation and perceived competence, through goal‐directed and cognitively demanding tasks within immersive environments (Güler et al. 2026; Lin et al. 2024). Furthermore, IVR scenarios can simulate environmental determinants associated with school participation, such as rule following, structured task demands, sensory stimuli and contextual cues (Maciver et al. 2019).

Existing evidence suggests that VR‐based interventions for ADHD have primarily targeted neurocognitive and symptom‐related outcomes, with systematic reviews reporting improvements in domains such as sustained attention/vigilance, memory and executive functioning (Romero‐Ayuso et al. 2021; Single et al. 2025). More recent syntheses similarly indicate potential benefits for executive functions and emotion regulation, while emphasizing the need for standardized outcomes and stronger trials (Sun et al. 2025). However, these studies rarely include direct measures of school participation outcomes (frequency and involvement) or explicitly examine the environmental and child determinants of school participation using school‐based outcome measures. In line with contemporary participation theory distinguishing participation outcomes (frequency and involvement) from participation‐related constructs, the SPQ was developed to provide information about environmental and child determinants rather than the amount/type of participation (Imms et al. 2016). Therefore, the present trial focused on teacher‐rated determinants in the school context as a feasible and theoretically aligned first step, while recognizing that future research should pair determinant measures with direct participation outcomes.

Prior IVR reviews and meta‐analyses have primarily emphasized neurocognitive or diagnostic outcomes—such as attention, vigilance, working memory and symptom ratings—as well as VR‐based classroom assessments. However, limited research has focused on school participation (frequency and involvement) or its underlying environmental and child‐based determinants (Romero‐Ayuso et al. 2021; Corrigan et al. 2023; Parsons et al. 2019; Neguț et al. 2017; Goharinejad et al. 2022). This study addresses that gap by evaluating an IVR intervention for children with ADHD on school participation–related constructs, focusing on the environmental and child determinants of school participation.

## 2 Material and Methods

Our study was evaluated by the University Noninvasive Clinical Research Ethics Committee and found to be ethically appropriate on 15.05.2024 (Decision Number: 09). Written informed consent was obtained from the parents of all participating children and from all participating teachers prior to enrollment. In addition, verbal assent was obtained from the children. The study procedures were explained in an age‐appropriate manner, and participation was entirely voluntary.

### 2.1 Participants

The study included children diagnosed with ADHD by a child psychiatrist according to DSM‐5 criteria who were receiving services at the university's paediatric occupational therapy unit and who voluntarily agreed to participate in the study. Inclusion criteria (applied to both intervention and control groups) were as follows: (1) ADHD diagnosis per DSM‐5; (2) primary school age; (3) informed, voluntary participation; and (4) enrollment in mainstream formal education. Exclusion criteria were as follows: (1) any additional neurodevelopmental condition (e.g., cerebral palsy and autism spectrum disorder); (2) receipt of other therapies during the study period (e.g., speech or physical therapy); and (3) home access to a VR headset, to prevent unscheduled exposure to the intervention game.

#### 2.1.1 Procedures

Ninety‐two participants were randomized 1:1 to intervention (*n* = 46) or control (*n* = 46) using an online randomization tool (https://www.randomizer.org/). The intervention group completed an IVR program twice weekly (45 min/session) for 8 weeks, delivered by the first author. The control group received no additional therapy during this period (waiting‐list control). To characterize and compare groups, the second author administered the Bruininks–Oseretsky Test of Motor Proficiency Test 2 Brief Form (BOT‐2 BF), and teachers completed the SPQ at baseline and post‐intervention to evaluate school participation–related outcomes. The BOT‐2 BF was administered at baseline to characterize participants' overall motor proficiency and to examine between‐group comparability in general motor competence; it was not used as an inclusion criterion. The purpose was not to ensure equivalence across each BOT‐2 subdomain, but to reduce the likelihood that marked differences in overall motor competence would confound the study findings. SPQ was completed by teachers working in public primary schools. Teachers were blinded to the group allocation of the children throughout the study period. After the trial and data analysis, 17 control participants who expressed interest were offered the IVR program irrespective of study results. No gift cards or other incentives were provided.

**FIGURE 1:**
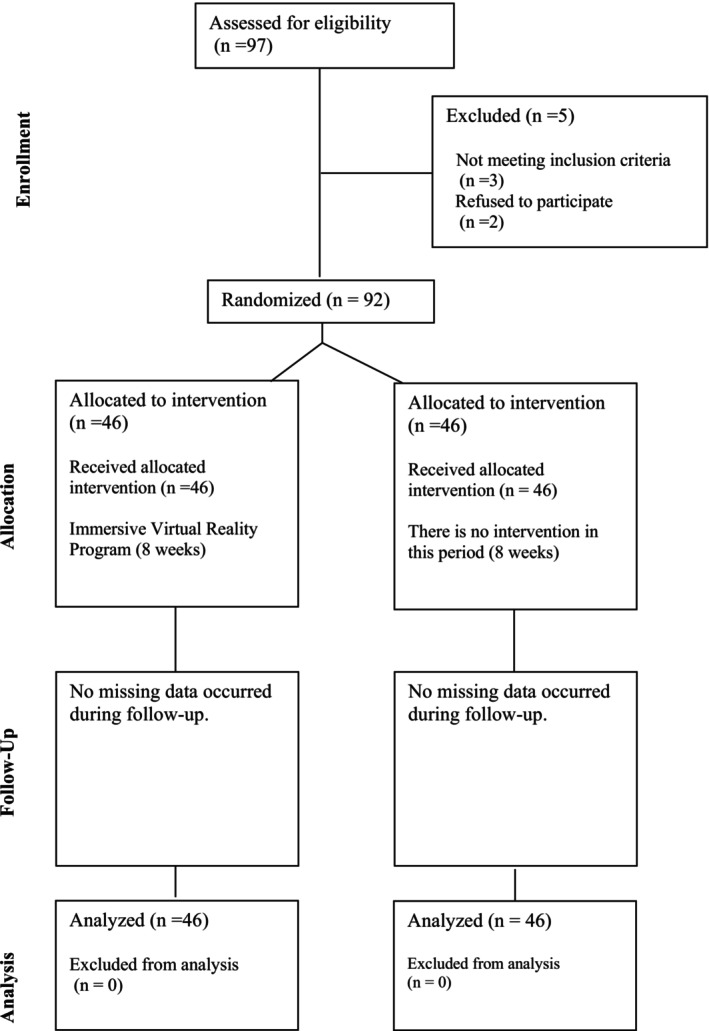
CONSORT diagram of the intervention participation process.

Figure 1 displays the CONSORT flow diagram of the intervention process (see Figure 1).

### 2.2 Measures

#### 2.2.1 Demographic Information Form

This form was used to obtain the demographic information of the children diagnosed with ADHD who participated in the study. This form collected information on age, gender, medication(s) used, treatments received, class and family education level.

#### 2.2.2 Bruininks–Oseretsky Test of Motor Proficiency Test 2 Brief Form

The BOT‐2 BF was designed to evaluate the fine and gross motor abilities in children between the ages of 4 and 21. The test is comprised of a total of 12 items and eight subtests. Subtests and items of BOT2‐BF are as follows: (1) Fine Motor Accuracy, (2) Fine Motor Integration, (3) Manual Dexterity, (4) Bilateral Coordination, (5) Balance, (6) Speed and Agility, (7) Coordination of Upper Extremities and (8) Endurance. The test is administered over a period of approximately 15–20 min. The maximum attainable score on this test is 72 (Bruininks 2010).

#### 2.2.3 School Participation Questionnaire

The SPQ comprises 44 items rated 1–4 (*disagree*–*agree*) with a two‐week reference period. It includes four scales: (1) the ‘competence’ or ‘doing’ subscale, consisting of 11 items, evaluates aspects such as choices, persistence, expectation fulfilment, role performance and skills. (2) the ‘mind and body experience’ or ‘symptoms’ subscale includes five items that gauge symptoms of low energy, fatigue, insomnia, pain, mood disturbances and anxiety. (3) The ‘identity’ or ‘being’ subscale, which comprises nine items, assesses the child's thoughts, feelings, knowledge, preferences, self‐perception and role perception. (4) the ‘Environment’ subscale contains 19 items that evaluate both the physical (e.g., places and objects) and social (e.g., peers, teachers and routines) aspects of the school environment. Total scores range from 44 to 176; higher values indicate more favourable environmental and child determinants of school participation. Psychometric evaluation of the original measure reported strong internal consistency (*α* = 0.84–0.94), acceptable Rasch reliability and adequate person separation (Maciver et al. 2020). The Turkish adaptation (T‐SPQ) demonstrated comparable reliability in children with ADHD (*α* = 0.85–0.91) (Kose et al. 2026).

### 2.3 Intervention

The immersive VR intervention was delivered with an Oculus Quest 2 headset using Job Simulator, a commercially available application that situates participants in a fictional ‘job museum’ staffed by robots. The program comprises four scenarios—office worker, gourmet chef, store clerk and auto mechanic—each organized as a sequence of tasks of increasing complexity. In Office Worker, participants completed 13 tasks spanning basic routines (e.g., eating breakfast) to typical office functions (e.g., managing phone calls and evaluating employees). Gourmet chef progressed from simple meal preparation (eggs and bacon) to 17 multistep culinary missions (e.g., crafting customized pizzas). In store clerk, players served customers by dispensing requested items (e.g., food, magazines and lottery tickets), preparing food and completing 16 structured tasks while also freely interacting with objects. Auto mechanic required selecting appropriate spare parts from multiple options, performing vehicle repairs and occasionally implementing unrequested modifications.

As the game advanced, both task difficulty and the level of instruction increased, requiring the use of executive functions such as sustained attention, planning and inhibitory control. Our rationale for designing the intervention from this perspective is based on participation frameworks, which identify child factors (e.g., competence, identity and regulation of mind and body) and environmental factors as central determinants of school participation (Imms et al. 2017; Maciver et al. 2019). Although we cannot assume that training executive skills in IVR will directly translate to greater school participation, these frameworks suggest that strengthening such determinants may increase children's capacity to manage school routines and respond to environmental demands. In addition, the intervention is structured as a game, which positions it as a leisure activity. From a participation perspective, engagement in leisure is itself meaningful and may foster motivation, enjoyment and a sense of identity (King et al. 2003). Thus, our approach integrates both direct participation (through leisure engagement) and potential indirect influences on participation (via underlying capacities), aligning with the theoretical understanding that participation is shaped by the dynamic interaction of child and environmental determinants rather than by skill acquisition alone.

In selecting job simulator, our aim was not to replicate classroom activities, but to provide a structured, goal‐directed virtual environment that systematically engages executive and self‐regulatory processes. The game requires sustained attention, task initiation, planning, inhibitory control and regulation across progressively demanding multistep scenarios. These characteristics align with child determinants emphasized in participation frameworks, particularly capacities and regulation processes relevant to managing everyday routines. Thus, the intervention was selected not to directly simulate school participation, but to target underlying determinants that may be pertinent to school functioning.

Over 8 weeks, participants completed the four scenarios in a fixed order with equal time per session; task order and duration were standardized. The intervention was conducted in a 16‐m^2^, furniture‐free therapy room within the university's paediatric occupational therapy unit, equipped with a 25‐cm cushioned floor to ensure safety. Prior to each session, children received a brief explanation of the game scenario and rules of the tasks. After this orientation, no additional guidance or performance‐related prompting was provided during the VR tasks.

Figure S1 showcases representative screenshots of the tasks included in the ‘Job Simulator’ video game (see Figure S1).

### 2.4 Statistical Analysis

Analyses were conducted in IBM SPSS Statistics Version 27 (Statistical Package for the Social Sciences). Categorical variables are reported as counts and percentages (*n* [%]) and continuous variables as mean (M) ± standard deviation (SD). Distributional assumptions were checked with the Shapiro–Wilk test (preintervention and postintervention); when normality held, we used parametric tests: chi‐square (χ^2^) tests for categorical baseline comparisons (e.g., gender and medication), independent‐samples *t‐*tests for between‐group differences (including continuous baseline variables such as age) and paired‐samples *t*‐tests for within‐group pre–post change. All tests were two‐tailed with an alpha (α) level of 0.05. Effect sizes were expressed as Cohen's *d* and interpreted as small (~0.2), medium (~0.5), large (~0.8) and very large (~1.3). To aid interpretation, profile plots depicting trajectories over time and group contrasts were generated in Python 3.10 using the Pandas, Matplotlib and Seaborn libraries.

## 3 Results

The mean age was 8.57 ± 1.50 years in the study group and 8.65 ± 1.39 years in the control group, with no statistically significant difference (*p* = 0.856, independent samples *t*‐test). The proportion of female participants was 58.6% in the study group and 65.2% in the control group, and the difference was not significant (*p* = 0.182, chi‐square test). Regarding medication use, 30.4% of the study group and 32.7% of the control group were using medication, again with no significant difference (*p* = 0.798, chi‐square test).

Table 1 outlines the demographic characteristics of the participants (see Table 1).

**TABLE 1:** Demographic characteristics of participants.

		Study group	Control group	*p*
Age (mean/standard deviation)		8.57/1.50	8.65/1.39	0.856*
		*n* (%)		
Gender	Female	27 (58.6)	30 (65.2)	0.182**
	Male	19 (41.4)	16 (34.8)	
Medication	Yes	14 (30.4)	15 (32.7)	0.798**
	No	32 (69.6)	31 (67.3)	

* Independent samples *t*‐test.

** Chi‐square test.

Table 2 provides the baseline comparison of BOT‐2 BF and SPQ scores. At baseline, the intervention and control groups did not differ on total school participation or on most BOT‐2 BF totals/subscales. The sole exception was Fine Motor Integration, where the intervention group scored higher than controls (mean difference = 0.76, SE = 0.263, *p* = 0.005). All other comparisons, including school participation subdomains (environmental factors, symptoms, competence and personality) and BOT‐2 BF indices (e.g., strength, balance and manual dexterity), were nonsignificant (*p* > 0.05), indicating satisfactory between‐group equivalence prior to the intervention (see Table 2).

**TABLE 2:** Baseline comparison of BOT‐2 BF and SPQ scores.

	Statistic	*p*	Mean difference	SE difference
SPQ total	0.2831	0.778	1.3043	4.607
Environment total	0.3292	0.743	0.7826	2.377
Symptoms total	−0.2486	0.804	−0.1739	0.700
Doing total	0.2114	0.833	0.3043	1.440
*Being total*	0.3834	0.702	0.3913	1.020
BOT‐2 total score	0.0631	0.950	0.0870	1.377
BOT‐2 strength	0.5307	0.597	0.1522	0.287
*BOT‐2 upper‐limb coordination*	−0.5796	0.564	−0.2609	0.450
BOT‐2 agility	−0.4427	0.659	−0.1522	0.344
BOT‐2 balance	−0.9121	0.364	−0.1957	0.214
BOT‐2 body coordination	0.2961	0.768	0.1087	0.367
BOT‐2 manual dexterity	−1.0000	0.320	−0.1522	0.152
Bot‐2 fine motor integration	2.8889	0.005	0.7609	0.263
BOT‐2 fine motor precision	−0.6202	0.537	−0.0652	0.105

Abbreviations: BOT2‐BF, Bruininks–Oseretsky Test of Motor Proficiency Brief Form; SE, Standard Error; SPQ, School Participation Questionnaire.

*p* < 0.05.

Table 3 compares the school participation scores between the groups. Within‐group analyses showed significant gains for the intervention group on all SPQ domains: SPQ total score rose from 136.6 ± 21.01 to 150.0 ± 14.45 (*p* < 0.001, *d* = 0.978), with parallel increases in environment (58.8 ± 11.26 → 64.5 ± 8.53), symptoms (15.3 ± 3.37 → 17.5 ± 2.46), doing (32.4 ± 6.31 → 36.0 ± 4.53) and being (30.0 ± 5.00 → 32.0 ± 3.44).

**TABLE 3:** Comparison of School Participation Questionnaire scores.

	Study group (*n* = 46)				Control group (*n* = 46)					
School Participation Questionnaire			Within group				Within group		Between groups	
	Before intervention M/SD	After intervention M/SD	*p*	*d*	Before intervention M/SD	After intervention M/SD	*p*	*d*	*p*	*d*
SPQ total	136.6/21.01	150.0/14.45	< 0.001	0.978	134.9/22.85	132.2/19.96	0.064	−0.326	< 0.001	1.165
Environment total	58.8/11.26	64.5/8.53	< 0.001	0.656	57.8/11.48	55/10.52	< 0.001	−0.792	< 0.001	0.969
Symptoms total	15.3/3.37	17.5/2.46	< 0.001	0.910	15.5/3.33	14.8/2.72	0.045	−0.305	< 0.001	1.031
Doing total	32.4/6.31	36/4.53	< 0.001	0.890	32/7.36	31/6.61	0.064	−0.326	< 0.001	0.878
*Being total*	30/5	32/3.44	0.004	0.452	29.6/4.74	28.2/4.23	< 0.001	−0.558	< 0.001	0.983

Abbreviations: M, mean; SD, Standard Deviation.

* *p* < 0.05.

** *p* < 0.005.

*** *d < 0.20 = small*.

**** *d* *= 0.50 medium*.

***** *d > 0.80 large effect size*.

Controls showed no change in SPQ total score (134.9 ± 22.85 → 132.2 ± 19.96) or doing and declines in environment (57.8 ± 11.48 → 55.0 ± 10.52), symptoms and being (29.6 ± 4.74 → 28.2 ± 4.23).

**FIGURE 2:**
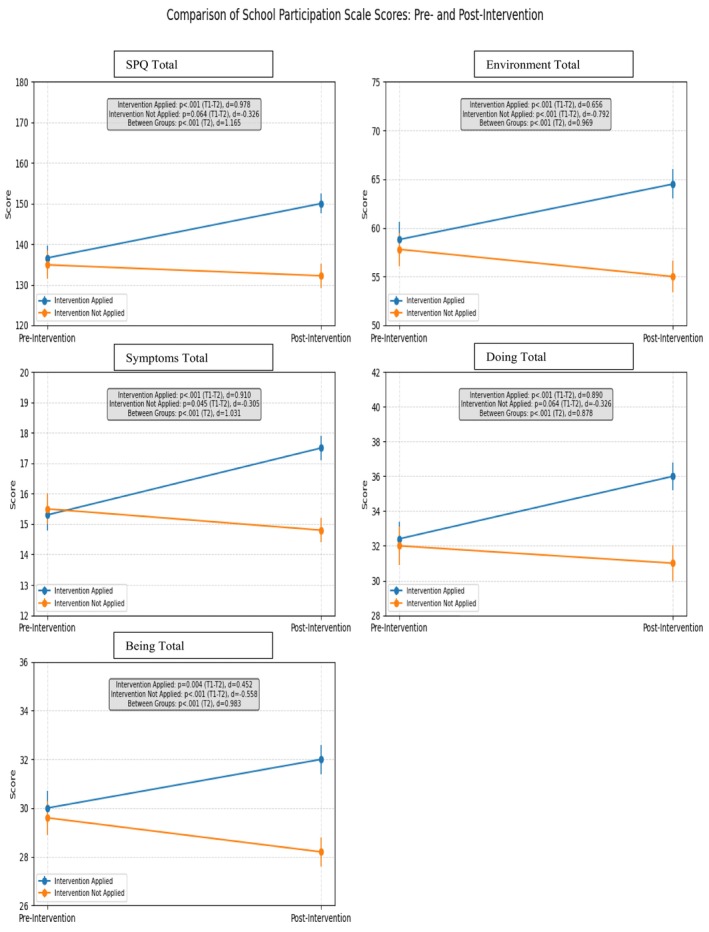
Comparison of SPQ scores: pre‐intervention and post‐intervention.

Posttest between‐group differences favoured the intervention across all domains, with large effects: SPQ total score (*d* = 1.165), environment (*d* = 0.969), symptoms (*d* = 1.031), doing (*d* = 0.878) and being (*d* = 0.983). Figure 2 illustrates the preintervention and postintervention comparison of SPQ scores (see Table 3 and Figure 2).

## 4 Discussion

These findings indicate that an 8‐week IVR program can enhance school‐focused participation‐related constructs in children with ADHD. Compared with controls, the IVR group showed significantly higher SPQ total and subscale scores, whereas controls declined. To our knowledge, this is among the first school‐context studies to evaluate IVR using a teacher‐rated tool explicitly targeting the environmental and child determinants of school participation, rather than diagnosis or symptoms alone. Interpreted within the fPRC framework (Imms et al. 2016; Imms et al. 2017), these gains may reflect changes in proximal determinants capacities, sense of self and use of environmental opportunities. Importantly, the SPQ assesses participation‐related constructs (determinants) rather than participation outcomes (frequency and involvement). The results extend prior VR–ADHD work by focusing on participation‐related constructs rather than solely executive functions or behaviours assessed outside natural school contexts (Wong et al. 2024; Romero‐Ayuso et al. 2024; Yu et al. 2024).

The being/identity domains of the SPQ capture teacher‐observed indicators of a child's sense of self within their school role, including their understanding of responsibilities, adherence to routines and capacity for realistic self‐appraisal (Maciver et al. 2020; Maciver et al. 2023). Converging evidence suggests that VR‐based interventions can enhance the competencies underpinning being/identity, particularly executive and self‐regulatory processes. For instance, recent systematic reviews report that VR‐based exercises improve inhibitory control, cognitive flexibility and working memory in youth with ADHD (Sun et al. 2025). Similarly, the SmartAction‐VR multitask paradigm, which simulates everyday activities, has demonstrated benefits in planning and goal‐directed problem solving, thereby linking executive functioning to ecologically valid performance. Furthermore, VR‐based learning interventions designed to boost academic motivation and on‐task behaviour have been found to sustain engagement during instructional activities (Alobidi et al. 2024). Consistent with this literature, our study observed a significant increase in being/identity scores among children who received the IVR intervention. This provides evidence of strengthened capacities and an enhanced sense of self, which are theoretically positioned to support long‐term participation in school routines (Maciver et al. 2022). In alignment with the fPRC's transactional framework, engaging in goal‐directed and structured IVR tasks may foster the development of self‐regulatory processes. Although the absence of performance‐based measures for executive functioning precludes an empirical evaluation of this underlying mechanism in the current study, it is plausible that the intervention facilitates individual gains in executive control and self‐regulation. These improvements may enable students to better anticipate classroom activities, adhere to routines and navigate environmental opportunities, thereby empowering reflective self‐appraisal within their academic roles (Imms et al. 2017; Maciver et al. 2023).

Doing/competence—a subdomain of the SPQ that indexes the environmental and child determinants of school participation—reflects core cognitive and behavioural components such as problem solving, decision making, adherence to classroom rules, planning and self‐management, alongside social interaction and the motor functions required for school activities (Maciver et al. 2022). Building on emerging evidence that VR can support motor development (Yu et al. 2024), social participation–relevant behaviours (Alobidi et al. 2024) and executive functions such as attention, problem solving and planning (Sun et al. 2025; Wong et al. 2024), our findings show a significant increase in doing/competence scores following the IVR program, consistent with the SPQ framework and the fPRC's view that gains in activity competence are proximal, theoretically upstream determinants of participation (frequency and involvement), rather than direct measures of participation itself (Imms et al. 2016; Imms et al. 2017; Maciver et al. 2023). In line with this model‐based interpretation, we infer that the IVR sessions offered graded, feedback‐rich opportunities to practice and consolidate executive control, motor coordination and socially oriented self‐regulation within meaningful roles (e.g., problem solving, responsibility‐taking and rule‐following). One potential explanation is that repeated exposure to structured, multistep tasks may have helped children practice task persistence and rule‐following in a motivating context. Nonetheless, because we did not include performance‐based measures of executive functions/problem‐solving, we cannot determine whether changes in these person‐level capacities occurred or mediated the observed SPQ changes.

Symptoms/experience of mind and body—encompassing physical and emotional states such as energy levels, pain, sleep–wake patterns, anxiety and mood—represents a key subdomain essential for successful school participation (Maciver et al. 2020). Recent evidence indicates that VR interventions can significantly enhance emotional regulation in children with ADHD. For instance, randomized controlled trials have reported marked improvements in parent‐rated emotional control and self‐regulation following VR‐based programs (Wong et al. 2024; Martin‐Moratinos et al. 2025). Systematic reviews similarly conclude that VR environments provide structured, feedback‐rich opportunities to practice pain and anger management and to strengthen emotional self‐management (Babu and Joseph 2025; Doulou et al. 2025). Furthermore, a pilot study by Carballo‐Marquez et al. (2025) demonstrated that VR‐based cognitive training effectively improved emotional control, reduced anxiety and enhanced overall well‐being (Carballo‐Marquez et al. 2025). Consistent with these findings, our study revealed a statistically significant improvement in the Symptoms/experience of mind and body subdomain after intervention. It is plausible that the IVR intervention's game‐based components, which require active motor competence and problem‐solving, contributed to this outcome. Such engagement may have increased physical activity and arousal levels, potentially leading to reduced perceived pain. Moreover, the realistic and immersive design of the game likely fostered higher motivation and interest, thereby improving mood and alleviating anxiety.

Following our IVR intervention, the environment subscale score increased significantly despite no direct modifications to the school environment; within the Person–Environment–Occupation–Performance and ICF‐CY frameworks, these teacher‐rated changes could indicate differences in how children utilize environmental opportunities at school, but the underlying person‐level processes cannot be determined given the absence of direct measurement (Bass et al. 2024; WHO 2007). According to the SPQ theoretical framework, the environment domain encompasses both the physical (spaces and objects) and social (peers, teachers and routines) aspects of the school (Maciver et al. 2022; Maciver et al. 2019). Our IVR intervention systematically intensified the practice of motor–cognitive problem solving, sustained attention, social communication and emotional self‐regulation through game‐based activities that provided graded task difficulty, real‐time feedback and a safe practice environment. Evidence from randomized controlled trials suggests that VR offers significant benefits for emotion regulation and self‐regulation in children with ADHD (Wong et al. 2024; Martin‐Moratinos et al. 2025) while supporting the executive functions necessary for planning and maintaining classroom tasks (Sun et al. 2025). Consequently, these child‐level gains are likely reflected in teacher observations as more independent use of classroom materials, increased engagement in peer interactions and better adherence to classroom routines. This is also consistent with prior psychometric research indicating that SPQ‐environment subdomains index teacher‐rated perceptions of the social, physical and organizational features of the school setting (Maciver et al. 2022; Maciver et al. 2020). Our findings align with this pattern: Following the IVR intervention, enhanced self‐efficacy and behavioural self‐regulation may have reduced the perceived impact of environmental barriers while increasing the utilization of environmental facilitators (Maciver et al. 2022; Bass et al. 2024; WHO 2007).

Based on our findings, we interpret the negative and statistically significant pre–post changes in SPQ total and subdomain scores within the control group as a reflection of rising contextual demands throughout the school term. Factors such as increasing academic expectations, cumulative fatigue and fluctuating motivation may tax person‐level performance—including self‐regulation, task organization and emotion regulation—with downstream effects on school participation–related constructs (Neguț et al. 2017; Goharinejad et al. 2022). Evidence specific to ADHD indicates lower school participation and a heightened sensitivity to environmental barriers, making a downward trajectory in the absence of additional support both expected and plausible (Kara et al. 2025; Bruininks 2010). Longitudinal research further links higher baseline ADHD symptoms to subsequent difficulties in peer interaction and social preference, suggesting that more structured school programs can mitigate academic declines. This implies a more negative natural course in unsupported groups (Maciver et al. 2020; Kose et al. 2026). From a measurement perspective, the SPQ being/identity and environment subdomains are designed to capture teacher‐observed fit with rules and routines, independent use of materials and interaction with social, physical and organizational features of school; therefore, fluctuations in these directly observable behaviours would be expected to appear on these subdomains that index the environmental and child determinants of school participation (Parsons et al. 2007; Parsons et al. 2019).

Two practical considerations emerged from implementation and outcome collection. First, IVR delivery was occasionally constrained by device‐related interruptions (intermittent overheating and two session‐ending shutdowns) and transient simulation sickness in a small number of participants, which required brief pauses and careful scheduling between sessions. Second, because the SPQ captures behavioural and emotional indicators linked to the environmental and child determinants of school participation rather than participation outcomes per se, future studies may benefit from combining SPQ ratings with complementary participation measures (e.g., PEM‐CY, Children's Assessment of Participation and Enjoyment/Preferences for Activities of Children [CAPE/PAC] and Child and Adolescent Scale of Participation [CASP]) and brief teacher/youth interviews to contextualize ratings and triangulate findings. These considerations are reflected in the limitations discussed below.

### 4.1 Limitations

The intervention was delivered in a university‐based paediatric occupational therapy unit to support safety, standardization and fidelity (e.g., a consistent physical setup and supervised administration). However, this delivery model also reflects real‐world constraints: integrating repeated VR sessions into everyday classroom routines may not be feasible in many schools without additional infrastructure, staff time and designated spaces. Accordingly, future work should evaluate implementation pathways (e.g., school‐based or hybrid delivery) that preserve safety and fidelity while increasing ecological alignment with classroom routines.

A further consideration relates to baseline motor comparability. The BOT‐2 Brief Form was administered at baseline to characterize participants' overall motor proficiency and to examine broad comparability in general motor competence, rather than to establish equivalence across each specific motor subdomain. Although a baseline difference was observed in Fine Motor Integration, overall motor proficiency was broadly comparable between groups, suggesting that this finding reflects variability in a specific motor domain rather than a generalized imbalance in motor competence. This point should nevertheless be considered when interpreting the findings.

Lastly, outcome assessment relied on teacher‐completed SPQ ratings obtained from public school contexts. Coordinating timely completion of school‐based measures across multiple classrooms required substantial communication and scheduling and constrained the extent to which we could include more resource‐intensive approaches (e.g., repeated classroom observation, multi‐informant interviews or additional school administrative data) within the study timeline. In this regard, the SPQ provided a feasible and school‐facing method for capturing the environmental and child determinants of school participation; nevertheless, future trials could strengthen inference by incorporating pragmatic triangulation strategies acceptable to schools (e.g., brief structured observation snapshots, complementary informant ratings and/or measures that distinguish participation outcomes such as frequency and involvement).

## 5 Conclusions

Compared to the control group, children with ADHD who participated in the IVR intervention demonstrated significantly greater gains in school participation–related constructs. These findings indicate that game‐based IVR can effectively enhance participation within the school context, likely by fostering higher levels of motivation and engagement. Overall, the results support the feasibility and potential utility of IVR as a valuable therapeutic adjunct in educational settings for children with ADHD.

## Author Contributions

**Barkın Köse:** writing – original draft, writing – review and editing, investigation, conceptualization, data curation. **Emine Cansu Güler:** writing – original draft, writing – review and editing, investigation, conceptualization, data curation. **Güleser Güney Yılmaz:** formal analysis, writing – review and editing, data curation. **Müberra Tanrıverdi:** data curation, formal analysis, methodology. **Dana Anaby:** writing – review and editing, supervision.

## Ethics Statement

The study was approved by the Noninvasive Clinical Research Ethics Committee of Bezmialem Vakıf University on 15 May 2024 (Decision Number: 09).

## Consent

All participants received information regarding the aims and procedures of the study and provided written informed consent before participation.

## Supporting information


**Figure S1:** Virtual reality simulation video game: Job Simulator.

## Data Availability

The data are not available due to ethical and confidentiality considerations.
