# Supplementary material for: Exploring Immersive Virtual Reality as an Approach to Improve School Participation–Related Constructs in Children With ADHD
Source: Child Care Health Dev. 2026 Aug 11;52(5):e70328. doi: 10.1111/cch.70328 (PMC13461272; doi:10.1111/cch.70328)
Supplement: Supplementary file 1 [file CCH-52-e70328-s001.docx]

**Supplementary Figure 1:** Virtual Reality Simulation Video Game: Job Simulator
